# A systemic review of maternal wellbeing and its relationship with maternal fetal attachment and early postpartum bonding

**DOI:** 10.1371/journal.pone.0220032

**Published:** 2019-07-25

**Authors:** Josephine McNamara, Michelle L. Townsend, Jane S. Herbert

**Affiliations:** 1 School of Psychology and Early Start, University of Wollongong, Wollongong, New South Wales, Australia; 2 Illawarra Health and Medical Research Institute, University of Wollongong, Wollongong, New South Wales, Australia; Monash University, AUSTRALIA

## Abstract

**Background:**

An emerging body of literature suggests there is a relationship between a pregnant woman’s psychological wellbeing and the development of maternal-fetal attachment (MFA) and early postpartum bonding. The nature of this relationship is not well understood because of the limited theoretical framework surrounding the construct of MFA and variations in study methods and data collection points. In this systematic review, we synthesize the published literature to determine the nature of the relationship from the antenatal to early postnatal period and to provide recommendations for future research and clinical practice.

**Method:**

Using the preferred reporting items for systematic reviews and meta-analyses (PRISMA) approach, four electronic databases were searched for peer-reviewed empirical studies, published in English. Articles were considered for inclusion if data was collected on at least one domain of maternal wellbeing/mental health and MFA during pregnancy or MFA during pregnancy and the mother-infant relationship during the early postpartum period (up to 12 weeks). No date parameters were applied to the search strategy. The review was registered with PROPSERO (registration number: CRD42018096174).

**Results:**

25 studies examining maternal mental health and MFA/postpartum bonding were selected for inclusion in this review. Key findings identified from the review were: a need to validate existing mental health measures or develop new measures specific for use in antenatal populations; inconsistencies in data collection points throughout pregnancy and postpartum; a lack of consensus about the construct of MFA and the way it is assessed; and a continued focus on postpartum outcomes.

**Conclusion:**

Scientific gaps remain in our understanding of the relationship between maternal mental health and both MFA and postpartum bonding which limit our theoretical understanding of the MFA construct. Recommendations for future research are to employ prospective longitudinal designs that span the full pregnancy and postpartum period, and for consistency in the terminology and methodology used when considering MFA. A re-focus of research attention on the theory behind MFA will allow a richer and more holistic account of the emerging relationship between mother and baby.

## Introduction

Pregnancy and the transition to parenting is a time of rapid physiological, psychological, and social change [1], which can be challenging and stressful for mothers [2–4]. International research shows that the antenatal period can be associated with increased distress and elevated psychological vulnerability [5], leaving women susceptible to mental health difficulties–that is, symptoms that cause significant distress and impair functioning [6]. Recent studies show that clinical indicators of depression, anxiety and stress are common during and after pregnancy [7–9], and that comorbid mental health symptomatology is prevalent [10–12]. These experiences may have a cumulative impact on an individual’s ability to balance psychological, social and physical resources with life challenges and stressors–a term referred to as ‘wellbeing’ [13]. Maternal distress has been found to be associated with poor obstetric outcomes [14–19] and impaired cognitive, behavioral and emotional child development [20–24]. Some studies have found that distress is higher during pregnancy than in the period following it [10, 25, 26], while other research suggests that a stable pattern of symptoms exists across the antenatal and postnatal periods [27]. Effective antenatal screening could both identify women with mental health problems during pregnancy and serve as a marker for those who may be at risk of continued distress post-childbirth.

During this period of transition and psychological vulnerability, the origins of the attachment relationship between a mother and her child begin to emerge [24, 28, 29]. It is well recognized that early attachment relationships play an important role in a child’s psychological, cognitive and social development [30, 31]. The attachment relationships individuals form with their primary caregivers during infancy and early childhood largely contribute to the way they interact with and relate to others in adulthood and the formation of their own attachment style [32, 33]. Research shows that parental mental illness during the early postpartum period may have negative effects on attachment formation, because of impairments in warmth, sensitivity and predictableness of parenting behaviors [34–36].

The term ‘maternal fetal attachment’ (MFA) describes the emotional bond between a mother and her unborn child during pregnancy [37]. Cranley [37] originally defined MFA as “the extent to which women engage in behaviors that represent an affiliation and interaction with their unborn child” (p282) and emphasized the establishment and strengthening of a unique relationship. Building on Cranley’s conceptualization, Müller proposed that the definition of MFA should also involve the thoughts and fantasies expectant mothers have in relation to their unborn baby and their pregnancy [38–42]. Conversely, Condon proposed that MFA was driven by a mother’s disposition to know, protect, interact with and meet the needs of her baby [43]. Despite the differences in definitions, theorists and researchers agree that MFA is a multi-dimensional construct that includes maternal thoughts, behaviors, emotions and attitudes [37, 44]. Although less researched than postpartum bonding, studies suggest that the experience of mental health difficulties antenatally may impair a mother’s ability to form a close bond with her unborn baby [45, 46]. Possible explanations for this include lack of emotional resources, beliefs about poor suitability and competence as a parent, lack of maternal role identity and negative attitudes towards caregiving [47–49].

Despite interest in early attachment relationships and the impact of maternal psychological health during this developmental stage, there continues to be contention as to the ‘best’ way to understand and categorize MFA [41]. The processes underlying MFA do not fit with traditional conceptualizations of attachment [50] as described by Bowlby [51] and Ainsworth [31]. The attachment system is described as the way a child seeks care, comfort and security from a caregiver, and the way a caregiver recognizes and responds to those needs (i.e. care-seeking and caregiving) [51]. MFA, however, is based on a mother’s attempts to love, care for and protect her child during pregnancy (i.e. caregiving only) [43]. While attachment involves a dyadic and reciprocal interaction, MFA is unidirectional [44, 52], a distinction which has resulted in a number of different terms being introduced to define the concept, including antenatal attachment [45], perinatal bonding [53] and emotional involvement [27]. Although the term ‘attachment’ is a poor fit, other commonly used phrases such as ‘bond’ and ‘relationship’ are also semantically incorrect [50]. This suggests the need for researchers to examine antenatal and postnatal experiences through different theoretical frameworks [52], and develop new concepts specifically for the pregnancy period. We acknowledge the limitations of the term MFA in this systematic review, but adopt it in the interest of consistency as it remains the most commonly used term in the literature.

The construct of MFA has been identified as an important contributor to mother and infant health [54], but the dominant focus of research has remained on postpartum mother-infant interactions [55, 56]. MFA research has considered a number of variables relating to wellbeing and mental health, including depression, anxiety, stress, coping skills, social support, partner relationships and self-concept [57, 58]. Although it has been the subject of research attention since the 1970s, across-study findings on MFA continue to be inconsistent [57, 59, 60], with previous reviews being unable to produce robust scientific findings [61]. Furthermore, despite recognition of the first 12 weeks after birth as a particularly critical time for mothers and infants–a period coined the ‘fourth trimester’–there remains a focus on studies with either an antenatal or postnatal focus [62, 63]. Inconsistencies in how maternal mental health and MFA are described and measured, and the lack of a single operational definition and theoretical framework underpinning MFA [43, 57, 58], represent two major gaps in the literature. Methodological decisions such as the primary use of cross-sectional designs has limited predictive abilities within studies, while disparity in assessment time points, small and homogenous samples, and variability in screening tools utilized has limited generalizability across studies [57, 59, 60]. Although reviews have drawn attention to these concerns, they have not served as a catalyst for future research that overcomes these weaknesses. Two recently published systematic reviews have attempted to address these concerns by examining MFA in relation to anxiety and child developmental outcomes [54, 64], however there remains a need to review studies on more global mental health constructs and maternal outcomes.

This systematic review aims to guide future research and clinical practice by examining the complex relationship between mental health, MFA, and early postpartum bonding from pregnancy to 12 weeks postpartum. The primary aim of this review is to investigate the relationship between maternal mental health and MFA. A secondary aim is to investigate the relationship between maternal mental health and postpartum bonding in studies which also examined MFA. By reviewing studies with both an antenatal and postnatal focus, we aim to provide a holistic account of the trajectory of experiences across the perinatal period. We seek to identify how maternal mental health and MFA are being described and measured in the literature, providing the first systematic review of MFA studies examining multiple domains of maternal mental health within the last 10 years. By recognizing the methodological limitations associated with MFA, and utilizing a robust systematic design, our overarching goal is to identify conclusions that can be drawn across study designs to understand the emerging relationship between mother and baby.

## Methods

### Protocol

**Fig 1:**
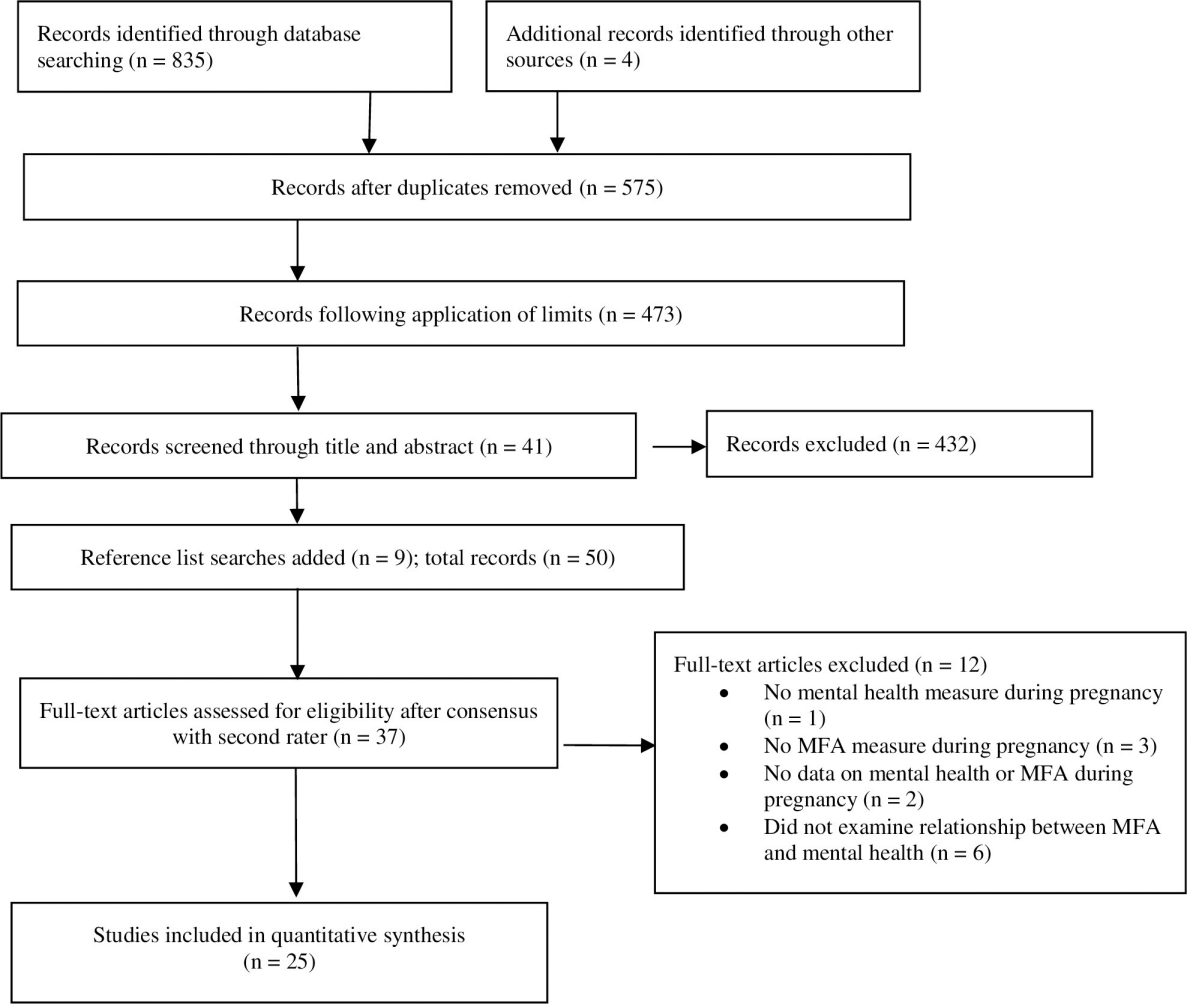
PRISMA flowchart for study identification and selection process.

The protocol for the current study was registered with the *International Prospective Register of Systematic Reviews* (PROSPERO, registration number: CRD42018096174). The search strategy used to identify articles for inclusion in the review was in accordance with the *Preferred Reporting Items for Systematic Review and Meta-Analysis* (PRISMA) guidelines for reviews (Fig 1) [65].

### Search strategy

Studies included in this review were identified by searching online databases and reference lists of identified articles between May and June 2018. An online database search was made of the following sources: PsycINFO, MedLine, CINAHL and Scopus. The search strategy incorporated three concepts: stage of pregnancy or postpartum period, maternal psychological health, and the developing emotional relationship between mother and fetus/infant. Search terms were: (pregnan* or antenatal or prenatal) AND (wellbeing or quality of life or mental health or psychiatric or distress or stress or depress* or anxi*) AND (attachment or bond*) AND (maternal f?etal or mother infant). No date parameters were placed on the search strategy. The search strategy included the terms “attachment” and “bond” to account for the variability in terminology observed within the existing MFA literature.

All records were imported into EndNote (Version X8). Articles considered for inclusion were limited to non-duplicated articles published in English in peer-reviewed journals. Inclusion and exclusion criteria were applied to remaining articles. Titles and abstracts were screened to identify studies with a focus on MFA and wellbeing/mental health during pregnancy and/or during pregnancy and the postpartum period. Review papers and studies examining the efficacy of an intervention were removed. The reference lists of articles being considered for review were searched to ascertain eligibility, and studies meeting inclusion criteria were added to the review. A second reviewer screened the identified titles and abstracts of articles considered for inclusion before a full-text review was completed. There was no disagreement on inclusion of articles.

### Study selection

Articles were considered for inclusion in the current review providing that they met the following eligibility criteria:

- Published in English within a peer-reviewed journal.
- Data collection took place during pregnancy and/or during pregnancy and the early postpartum period (i.e. up to 12 weeks).
- Focus on maternal outcomes (i.e. not infant outcomes alone).
- Measures were included to assess MFA and at least one domain of wellbeing or mental health (e.g. depression, anxiety, stress).
- Participants were female and aged 16 years and over.
- Studies were quantitative (i.e. not exclusively qualitative).
- The purpose of the study was not to evaluate the efficacy of an intervention.

A decision was made to include studies that collected data from participants during pregnancy and up to 12 weeks postpartum. This early postpartum period has been recognized as a critical time for mothers and infants [62, 66] because of the vulnerability of mothers’ mental health [67] and intensive caregiving duties required for newborns [63, 68].

We applied an inclusion criterion of participants aged 16 years and over because this is the recommended age for minimal risk research [69]. We acknowledge that there are competing positions on the appropriate minimum age for research participation [70], with 18 years being the legal age of informed consent [71]] and 20 as the start of adulthood as defined by the World Health Organization [72]. Thus, our inclusion criterion may capture publications excluded from previous reviews [27, 73].

We chose to exclude intervention studies from this review because our primary focus was to identify whether an association existed between mental health and MFA/postpartum bonding without the influence of and exposure to a treatment, program, or other type of intervention. This decision was made in consultation with other reviews within the field [74, 75].

### Quality assessment

A formal assessment of article quality was performed by two members of the research team independently using the *Appraisal of Cross-Sectional Studies* (AXIS) [76]. AXIS is a quality assessment tool designed to assist researchers to critically appraise studies, specifically in the process of conducting a systematic review. The tool was developed in consultation with current literature and the recommendations of a Delphi panel of research experts [76]. Although the measure was originally developed for cross-sectional studies, the 20 items pertaining to the identification of focused research aims, appropriateness of study design, use of valid measures and statistical analyses and consideration of bias, were relevant for both the cross-sectional and longitudinal studies included in the current review. The checklist design of the AXIS does not provide a cut-off numerical score for study eligibility. Instead it allows users the flexibility of a subjective assessment of overall quality and encourages consideration risk of bias and quality of reporting for each component of the study design–a feature other quality assessment tools do not allow [77, 78]. Given its recent publication (2016), the AXIS has not yet been validated. Despite these limitations, there is currently no gold standard tool for assessing the quality of observational studies [79]. Therefore, using a newly developed tool that attempts to address the shortcomings in other available tools is justified.

### Data extraction

Following quality assessment, the first author extracted information from included studies pertaining to study aims, participant information, study design, assessment time points, location, measures, data analyses and key results. This process was overseen by a second researcher within the research team.

## Results

### Literature search

A total of 839 articles were identified by electronic database searching (n = 835) and additional records known to authors (n = 4). After removing duplicates (n = 264) and articles not published in English or peer-reviewed (n = 102), 473 studies remained for screening. Articles were screened by title and abstract to identify empirical studies with a focus on MFA and wellbeing or mental health during pregnancy or during pregnancy and in the first 12 weeks after childbirth (n = 41). A manual search was made of the reference list of each included article, which resulted in an additional nine articles being added to the review (n = 9). No further appropriate studies were found when searching the reference lists of the nine additional articles. The remaining papers were screened by a second reviewer before being assessed for full-text eligibility (n = 50). Based on their abstracts, a total of 37 articles appeared to meet inclusion criteria and were included in the full-text review. Following discussion between reviewers, 12 studies were excluded, in accordance with eligibility criteria, leaving 25 articles for inclusion in the final review. This process is illustrated in Fig 1.

To determine the quality of the articles, the first and second researchers independently completed the AXIS for the 25 remaining studies. No numerical cut-off value is required by the AXIS, however articles which met fewer criteria should be interpreted with caution (Table 1). All studies met at least 11 of the 20 criteria. Twenty-four of the studies did not provide a justification of sample size, and four did not identify any study limitations. Four studies undertook measures to address and describe non-responders. One study used a sample that was not representative of the pregnancy population (i.e. recruited from a maternity shop) [80]. One study made reference to the use of the *Pregnancy Related Anxiety Scale* (PRAS) within the abstract of the paper, however no findings were reported in the methods or results section pertaining to the PRAS [81].

**Table 1:** AXIS quality assessment appraisal for studies included in the systematic review.

		1	2	3	4	5	6	7	8	9	10	11	12	13	14	15	16	17	18	19	20	21	22	23	24	25
Introduction	Clear aims	✓	✓	✓	✓	✓	✓	✓	✓	✓	✓	✓	✓	✓	✓	✓	✓	✓	✓	✓	✓	✓	✓	✓	✓	✓
Methods	Appropriate design	✓	✓	✓	✓	✓	✓	✓	✓	✓	✓	✓	✓	✓	✓	✓	✓	✓	✓	✓	✓	✓	✓	✓	✓	✓
	Sample size justified													✓												
	Population defined	✓	✓	✓	✓	✓	✓	✓	✓	✓	✓	✓	✓	✓	✓	✓	✓	✓	✓	✓	✓	✓	✓	✓	✓	✓
	Sample representative of population	✓	✓	✓	✓	✓	✓	✓	✓	✓	✓	✓	✓	✓	✓	✓	✓	✓	✓	✓	✓	✓	✓	✓	✓	✓
	Selection process representative	✓	✓	✓	✓	✓	✓	✓	✓	✓	✓	✓		✓	✓	✓	✓	✓	✓	✓	✓	✓	✓	✓	✓	✓
	Measures to address non-responders																				✓		✓	✓		✓
	Appropriate outcome variables	✓	✓	✓	✓	✓	✓	✓	✓	✓	✓	✓	✓	✓	✓	✓	✓	✓	✓	✓	✓	✓	✓	✓	✓	✓
	Valid measures	✓	✓	✓	✓	✓	✓	✓	✓	✓	✓	✓	✓	✓	✓	✓	✓	✓	✓	✓	✓	✓	✓	✓	✓	✓
	Defined statistical significance	✓	✓	✓	✓	✓	✓	✓	✓	✓	✓	✓	✓	✓	✓	✓	✓	✓	✓	✓	✓	✓	✓	✓	✓	✓
	Methods described	✓	✓	✓		✓	✓	✓	✓	✓	✓	✓	✓	✓	✓	✓	✓	✓	✓	✓	✓	✓	✓	✓	✓	✓
Results	Result data described	✓	✓	✓	✓	✓	✓	✓	✓	✓	✓	✓	✓	✓	✓	✓	✓	✓	✓	✓	✓	✓	✓	✓	✓	✓
	Concerns about non-response bias																									
	Non-responder information described							✓													✓		✓	✓		✓
	Results internally consistent	✓	✓	✓	✓	✓	✓	✓	✓	✓	✓	✓	✓	✓	✓	✓	✓	✓	✓	✓	✓	✓	✓	✓	✓	✓
	Results presented for analyses	✓	✓	✓	✓	✓	✓	✓	✓	✓	✓	✓	✓	✓	✓	✓	✓	✓	✓	✓	✓	✓	✓	✓	✓	✓
Discussion	Conclusions justified	✓	✓	✓	✓	✓	✓	✓	✓	✓	✓	✓	✓	✓	✓	✓	✓	✓	✓	✓	✓	✓	✓	✓	✓	✓
	Limitations identified	✓	✓	✓	✓	✓			✓	✓	✓			✓	✓	✓	✓	✓	✓	✓	✓	✓	✓	✓	✓	✓
Other	Funding sources or conflicts of interests																									
	Ethical approval/ consent attained	✓	✓	✓	✓	✓	✓	✓	✓	✓	✓		✓	✓	✓	✓	✓	✓	✓	✓	✓	✓	✓	✓	✓	✓

### Overview of included studies

In total, 25 of the originally identified 839 articles were included in the systematic review. All papers contained original quantitative data and were observational in nature. A total of 5983 female participants were included and participant ages ranged from 16–45 years. The characteristics of these studies are shown in *Table 2.* Thirteen of the articles employed a cross-sectional design and 12 were prospective longitudinal studies. All studies collected data during pregnancy, and six also followed women into the postpartum period. Publication dates ranged from 1997 to 2018. Sample sizes ranged from 30–751 (M = 239.28, SD = 184.49). There was no observed pattern in sample size based on location of publication. The majority of studies included participants from community samples, with the exception of three studies who utilized clinical populations (i.e. diagnoses of Major Depressive Disorder, hospitalized for pregnancy-related problems, and pregnancy as the result of IVF). Additional participant characteristics included women from lower socioeconomic backgrounds and of primiparous and multiparous status. Outcome variables included depression (n = 21), anxiety (n = 10), stress (n = 4), intimate partner/couple relationship (n = 6), social support (n = 7), wellbeing (n = 2), distress (n = 1), body dissatisfaction (n = 1), disordered eating (n = 1) and depressive rumination (n = 1). All studies employed self-report measures (n = 25), with one study additionally including observational measures (interview and clinician rated measure). A number of screening tools and assessment measures were used across the studies of which a summary is reported in *Table 3.* Across the 25 studies, 12 different measures were used to assess MFA and three measures were used to assess postpartum bonding. The most commonly used measure of MFA was the *Maternal Fetal Attachment Scale* (MFAS; n = 8), followed by the *Maternal Antenatal Attachment Scale* (MAAS; n = 7). The *Edinburgh Postnatal Depression Scale* (EPDS; n = 11) was the most used screening tool for depression.

**Table 2:** Overview of included studies.

		N	%
Study design	Cross-sectional	13	52
	Longitudinal	12	48
Data collection points (for longitudinal studies)	Two	8	32
	Three	3	12
	Four	1	4
Variables	Depression	21	84
	Anxiety	10	40
	Stress	3	12
	Other	13	52
Measure	Self-report Observational	25 1	100 4
Location	Asia Australia	9 2	36 8
	Europe	9	36
	North America	5	20

**Table 3:** Screening tools and measures of the studies included in the systematic review.

Variable	Measure	Acronym	Number
MFA	Maternal Fetal Attachment Scale	MFAS	8
	Maternal Antenatal Attachment Scale	MAAS	7
	Childbearing Attitude Questionnaire	CCAQ	1
	Mother-Infant Bonding Questionnaire	MIBQ	2
	Mother-to-Infant Bonding Scale	MIBS	2
	Modified Maternal Fetal Attachment Scale	MMFAS	2
	Awareness of Foetus Scale	AFS	1
	Antenatal Maternal Attachment Scale	AMAS	1
	Maternal Attitudes Questionnaire	MAQ	1
	Prenatal Attachment Inventory	PAI	1
	Prenatal Attachment Inventory Revised	PAI-R	1
	Parental Bonding Instrumental	PBI	1
Postpartum bonding	Mother-Infant Bonding Questionnaire	MIBQ	2
	Mother-to-Infant Bonding Scale	MIBS	2
	Postpartum Bonding Questionnaire	PBQ	1
Depression	Edinburgh Postnatal Depression Scale	EPDS	11
	Centre for Epidemiologic Studies Depression Scale	CES-D	3
	Zung Self-Rating Depression Scale	ZSDS/ZUNG	2
	Hamilton Rating Scale for Depression	HRSD	1
	Profile of Mood States	POMS	1
Anxiety	State Trait Anxiety Inventory	STAI	4
	Pregnancy‐Related Anxiety Scale	PRAS	1
	Penn State Worry Questionnaire-Past Week	PSWQ-PW	1
Stress	Pregnancy Stress Rating Scale	PSRS	2
	Life Events Scale	LES	1
	Prenatal Coping Inventory	PCI	1
	Prenatal Distress Questionnaire	PDQ	1
	Ways of Coping Checklist	WCC	1
Couple relationship	Dyadic Adjustment Scale	DAS	2
	Intimate Bond Measure	IBM	1
	Questionnaire on Partnership	PFB	1
Social support	Interpersonal Support Evaluation List	ISEL	1
	Japanese Social Support Questionnaire	J-SSQ	1
	Prenatal Psychosocial Profile	PPP	1
	Short Form Social Support Questionnaire	SSQ6	1
	Social Support Apgar	SSA	1
	Social Support Questionnaire	SSQ	1
	Social Support Scale	F-SozU-K-14	1
Combined measures	Hospital Anxiety Depression Scale	HADS	4
	Depression, Anxiety and Stress Scale Mental Health Inventory	DASS-21 MHI	1 1
Other	Body Shape Questionnaire	BSQ-R-10	1
	Chinese Childbearing Attitude Questionnaire	CCAQ	1
	Health Practices Questionnaire	HPQ	1
	Ruminative Response Scale	RRS	1
	Symptoms Checklist	SC	1
Interviews	Hollingshead Index of Social Status Interview	HISS	1
	Structured Clinical Interview for DSM-IV-TR	SCID	1
	Timeline Follow Back Interview	TLFB	1

The construct used to describe the emotional bond between mother and baby during pregnancy was primarily referred to as MFA (n = 14), but also included prenatal attachment (n = 4), perinatal bonding (n = 3), antenatal attachment (n = 1), maternal attachment (n = 1), maternal-fetal bonding (n = 1), and emotional involvement (n = 1).

A summary of the characteristics and results of the studies included in the systematic review are presented in Table 4.

**Table 4:** Characteristics and results of the studies included in the systematic review.

Author/s	Aims	Design	Location	Sample	N	MFA or bonding measure/s	Other measure/s	Key results
1. Alhusen et al., 2012* [73]	To investigate the influence of maternal depressive symptoms on MFA in a sample of low-income women.*	Cross-sectional *24–28 weeks*	US	Low-income	166	MFAS	EPDS PPP	Stronger MFA was correlated with lower depression and higher social support. Depressive symptoms and social support were significant predictors of MFA.
2. Barone et al., 2014* [82]	To examine the role of gestational age, couple adjustment and depressive symptoms on MFA in a sample of suburban women.*	Cross-sectional *9–41 weeks*	Italy	Low-risk; suburban	130	PAI	CES-D DAS	MFA was higher for mothers with higher perceived couple adjustment. Depression scores did not predict total MFA. Higher scores on the fantasy and sensitivity subscales (i.e. non-positive thoughts and feelings) of the PAI correlated with higher endorsement of depressive symptoms.
3. Chang et al., 2016 [83]	To explore the predictors of psychosocial stress during pregnancy.	Cross-sectional *Second or third trimester*	Taiwan	Low-risk	300	MMFAS	EPDS ISEL PSQI PSRS	Positive correlations were found between pregnancy stress and both depression and MFA. MFA and primiparous status were found to be predictors of pregnancy stress.
4. Condon & Corkindale, 1997 [45]	To examine the correlates of MFA in the third trimester of pregnancy.	Cross-sectional *>28 weeks*	Australia	Community	238	MAAS PBI	HADS IBM LES POMS SSQ ZSDS	Women with poorer MFA showed higher depression and anxiety, lower social support and higher control/domination/criticism within the intimate partner relationship. A negative association was found between MFA (MAAS-total) and depression on all measures except ZSDS. MFA quality was negatively correlated with all depression measures, while MFA intensity was negatively correlated with HAD-D only.
5. Doster et al., 2018* [81]	To investigate the relationship between MFA and postpartum bonding, with anxiety, depression and partner relationship.*	Longitudinal *T1*: *third trimester* *T2*: 5 weeks PP*	Germany	Community	324	MFAS (T1) PBQ (T2)	EPDS STAI (all T1-T2) PFB (T1)	Higher MFA was positively correlated with partner relationship quality, but not anxiety or depression. Stronger postpartum bonding was associated with lower state and trait anxiety, but not depression. Higher MFA was positively correlated with postpartum bonding.
6. Figueiredo & Costa, 2009 [27]	To examine the relationship between maternal prenatal and postnatal stress, mood and emotional involvement with the infant.	Longitudinal *T1*: *6 months* *T2*: *3 months PP*	Portugal	Primiparous	91	MIBS (T1-2)	EPDS STAI (all T1-2)	Depression predicted weaker MFA during pregnancy and poorer bonding postpartum, while anxiety predicted weaker bonding after birth only. Lower MFA predicted poorer emotional involvement with the infant and higher depression and anxiety at three months postpartum.
7. Goecke et al., 2012* [84]	To examine the relationships between MFA, perinatal factors and depression during pregnancy and postpartum (up to 18 months) in a sample of first-time mothers.	Longitudinal *T1*: *third trimester* *T2*: 3 weeks PP*	Germany	Primiparous	161	MAAS (T1)	EPDS (T1-2)	A negative correlation was found between MFA quality and depression during pregnancy, and MFA quality/global scores and depression at three weeks postpartum. Higher subjective wellbeing (as measured on a 1–5 Likert scale by participants at T1) was associated with stronger MFA global and quality scores during pregnancy. The intensity of MFA was not associated with depression or wellbeing.
8. Haedt & Keel, 2007 [85]	To investigate the relationship between MFA, depression and body dissatisfaction during pregnancy.	Cross-sectional *2–40 weeks*	US	Community	196	MFAS	BSQ-R-10 EPDS	No correlations were found between MFA and either body dissatisfaction or depression. Body dissatisfaction moderated the association between MFA and gestational age, but not depression. Greater gestational age predicted stronger MFA in women with low body dissatisfaction.
9. Hart & McMahon, 2006 [86]	To investigate the relationship between anxiety, depression and psychological adjustment to pregnancy.	Cross-sectional *20–28 weeks*	Australia	Primiparous	53	CAQ MAAS MAQ	EPDS STAI	Higher anxiety was correlated with lower MFA quality and more negative attitudes towards motherhood and the self as mother (i.e. higher maternal worries, more maladaptive cognitions about motherhood), but not MFA intensity or global scores (as measured by MAAS). No significant correlations were found between depression and MFA. Women who reported a negative quality of MFA showed higher symptoms of depression, trait anxiety and state anxiety.
10. Honjo et al., 2003 [87]	To examine the relationship between MFA and depression in first and second trimesters of pregnancy.	Cross-sectional *First or second trimester*	Japan	Community	216	AMAS	ZSDS	A positive correlation was found between MFA and number of social supports. No correlation was observed between MFA and depression.
11. Hsu & Chen, 2001 [88]	To investigate the relationship between pregnancy-specific and life-event stress with MFA.	Cross-sectional *>28 weeks*	Taiwan	Community	150	MMFAS	PSRS ACSEAL	Stronger MFA was associated with higher pregnancy-specific stress and lower life stress. Predictors of MFA included pregnancy-specific stress, life-event stress, parity and attendance at prenatal classes.
12. Kunkel & Doan, 2003* [80]	To investigate the relationship between MFA and depression.	Cross-sectional *During pregnancy *	Canada	Community	35*	MAAS MFAS	CES-D	Higher depression scores were associated with lower MAAS-quality and MAAS-global scores. No association was observed between MFAS total score or MAAS-intensity and depression.
13. Kuo et al., 2013 [89]	To investigate MFA throughout pregnancy in a sample of Taiwanese women who conceived through IVF.	Longitudinal *T1*: *9 weeks* *T2*: *12 weeks* *T3*: *20 weeks*	Taiwan	Primiparous; conceived through IVF	160	AFS MFAS (all T1-3)	CCAQ PRAS SC SSA (all T1-3)	Childbearing attitude, awareness of fetus and social support were predictors of MFA when gestational age was controlled for.
14. Lai et al., 2006* [90]	To examine the prevalence and psychosocial factors of disordered eating in new mothers.	Longitudinal *T1*: during pregnancy*	Hong Kong	Community	131	MPAS	EDI-2 GHQ SSS	Prenatal disordered eating was not correlated with MFA. Stronger MFA was correlated with higher instrumental and emotional spousal support.
15. Lindgren, 2001 [91]	To investigate the influence of depression on positive health practices directly and through MFA.	Cross-sectional *20–40 weeks*	US	Community	252	MFAS	CES-D HPQ	No correlation was found between depression and MFA. Higher depression and lower MFA were associated with fewer positive health practices. Higher depression was found to be a predictor of lower MFA.
16. Mako & Deak, 2014* [92]	To analyse MFA in relation to mental health, partner relationship, demographic and pregnancy variables.*	Cross-sectional *7–40 weeks*	Hungary	Community	237	MAAS*	DAS HADS	Higher MFA was correlated with lower anxiety and depression, and higher relationship adjustment, but not relationship length. MFA total and intensity scores (as measured by the MAAS) were higher in women who had detected fetal movement than those who had not yet detected fetal movement.
17. McFarland et al., 2011 [93]	To compare MFA in women with and without Major Depressive Disorder.	Longitudinal *T1*: *26 weeks* *T2*: *36 weeks*	US	With or without Major Depressive Disorder	161 (65 with MDD)	MFAS (T1-2)	HISS (T1) HRSD SCID TLFB (all T1-2)	Women with MDD had significantly lower MFA than women in the non-MDD group. Neither anxiety nor antidepressant use were associated with MFA. An inverse relationship was observed between depression severity and MFA and when considering the interaction of the MDD group and depression severity with MFA.
18. Mikulincer & Florian, 1999 [94]	To investigate the role of attachment style in bonding to the fetus, mental health and coping with pregnancy-related problems.*	Longitudinal *T1*: *7–12 weeks* *T2*: *22–24 weeks* *T3*: *32–34 weeks*	Israel	Primiparous; low-risk	30	MFAS (T1-3)	ASS MHI WCC (all T1-3)	Greater MFA was correlated with higher wellbeing and tendency to seek support, and lower distress and use of emotion-focused coping at T1. No patterns were observed for problem-focused or distance coping at T1. No significant associations were found between MFA and mental health variables at T2 or T3.
19. Ohara et al., 2017a [53]	To investigate the relationships between perinatal bonding failure, depression and social support among mothers.	Longitudinal *T1*: *<25 weeks* *T2*: *1 month PP*	Japan	Community	494	MIBQ (T1-2)	EPDS (T1-2) J-SSQ (T1)	Fewer supportive people during pregnancy predicted lower MFA and postpartum bonding and higher depression at both time points. Higher MFA was correlated with lower depression at T1. Similarly, higher bonding was correlated lower depression postpartum.
20. Ohara et al., 2017b [95]	To investigate the relationship between maternal depression and bonding failure during pregnancy and in the postpartum period.	Longitudinal *T1*:*<25 weeks* *T2*: *36 weeks* *T3*: *5 days PP*	Japan	Community	751	MIBQ (T1-3)	EPDS (T1-3)	Higher MFA was correlated with lower depression in early and late pregnancy (excluding anxiety and lack of affection at T1). Similarly, higher depression was associated with lower bonding postpartum. MFA predicted depressed mood at T2 and T3, but not at T1. Depression scores did not predict MFA scores.
21. Ohoka et al., 2014 [96]	To investigate the association between bonding disorder and maternal mood during pregnancy and in the postpartum period.	Longitudinal *T1*:*<25 weeks* *T2*: *36 weeks* *T3*: *5 days PP* *T4*: *1 month PP*	Japan	Community	389	MIBS (T1-4)	EPDS (T1-4)	Depression and MFA scores were correlated at T1-T4, with women reporting higher depressive symptoms having lower MFA and postpartum bonding. Women who reported continuous depressive symptoms over the testing points also showed sustained bonding difficulties.
22. Rubertsson et al., 2015 [46]	To examine the relationship between MFA with emotional wellbeing and obstetric, demographic and social factors.	Longitudinal *T1*: *8–10 weeks* *T2*: *36 weeks*	Sweden	Community	718	PAI-R (T1-2)	HADS (T1-2)	Higher depression scores were associated with lower MFA across the three PAI-R subscales. Higher anxiety was associated with higher PAI-R-Anticipation but not Interaction or Differentiation scores. Lack of perceived partner support was correlated with PAI-R-Interaction scores, while lack of perceived partner support was correlated with lower MFA on all subscales. Women who reported fewer positive feelings about birth and the early postpartum period during their pregnancy also reported lower MFA.
23. Schmidt et al., 2016 [97]	To determine whether depressive rumination and worrying are predictive of depressive and anxious symptomatology and MFA during pregnancy in a non-clinical sample.	Longitudinal *T1*: *1–20 weeks* *T2*: *21–40 weeks*	Germany	Community	215	MAAS (T1-2)	DASS-21 FSozUK14 PSWQ-PW RRS (T1-2)	Lower depressive rumination and higher social support were correlated with greater MFA. Depressive rumination at T1 was predictive of MFA intensity but not MFA quality at T2. Worry at T1 was not predictive of MFA at T2. Social support at T1 was predictive of MFA quality and intensity at T2.
24. Seimyr et al., 2009* [98]	To investigate how mothers and fathers think and feel about their babies, how parental-fetal attachment (PFA) is related to maternal depressive mood and the relationship between maternal mood and MFA.	Cross-sectional *30–32 weeks*	Sweden	Community	298*	MFAS	EPDS*	Women in the high depression group showed greater sensitivity to fetal movements (MFAS-IV) and less positivity towards the pregnancy and associated body changes (MFAS-III). No correlation was observed between depression and MFAS total score, or the remaining three subscales.
25. White et al., 2008 [99]	To model the relationships between maternal perceptions and medical ratings of risk, coping, psychological wellbeing and MFA in a sample of women hospitalised for pregnancy-related complications.	Cross-sectional *>23 weeks*	Northern Ireland	Hospitalized for pregnancy-related reasons	87	MAAS	HADS PCI PDQ SSQ6 STAI	Quality of MFA was positively correlated with history of anxiety/depression, positive appraisal and appraisal of own/baby’s health, and negatively correlated with current anxiety/depression and avoidance. Intensity of MFA was positively correlated with preparation, positive appraisal and appraisal of own/baby’s health, and negatively correlated with unplanned pregnancy, depression and avoidance. Positive appraisal (as a coping strategy) mediated the association between maternal appraisal of risk and MFA. HADS-anxiety was predictive of MFA intensity. Social support was not associated with MFA.

*Denotes missing information not relevant to the current review (including additional time points and participant groups outside of the parameters set for this review)

### Statistical analyses

The majority of papers used Pearson product-moment correlations (n = 22) and regression analyses (n = 15) for the purpose of statistical analyses. Structural equation modelling (n = 2), generalized linear models (n = 2), discriminant function analysis (n = 1), ANOVA (n = 5) and chi-square (n = 4) analyses were also utilized. Although the use of correlation analyses has remained consistent over time, the more recent studies included within the review were noted to employ more advanced statistical techniques [53, 89, 93, 95].

#### Main findingsDepression and MFA

Nineteen of the 21 studies investigating depression examined the relationship between depression and MFA (*note*: 82 and 97 did not), including the two studies with a sample of younger mothers (minimum age of 16 years). Higher depression was associated with lower MFA in the majority of publications [27, 46, 53, 73, 91–93, 95, 96]. These findings suggest that maternal mood negatively impacts on a mother’s ability to form an attachment to her unborn baby [45, 86] and may contribute to a sense of detachment [45]. However, four studies reported no relationship between depression and MFA [81, 85–87]. Furthermore, three studies found that depression was not a predictor of MFA [82, 95, 98]. Consistent with the idea of the changing nature of MFA, one study found that MFA predicted depression in late pregnancy and the early postpartum period but not in early pregnancy [95]. McFarland et al. [93] examined whether the severity of depression impacted MFA, and found that women with more severe MDD had poorer MFA than women with less severe MDD and those in the non-MDD group. In Schmidt et al.’s [97] study of depressive rumination in relation to MFA, a negative correlation was reported between the quality of the MFA in the first and second half of pregnancy, but no relationship with the intensity of the MFA. The authors suggested that perseverative thinking may reduce a mother’s available cognitive resources and contribute to limiting thinking about her unborn baby, thus having a negative effect on the development of MFA [46].

When considering MFA as measured at a subscale level, a number of studies found mixed results. Condon and Corkindale [45] found a negative correlation between EPDS and MAAS-quality/global, ZSDS and MAAS-quality, HAD-D and MAAS-quality/intensity/global, and POMS-D and MAAS-global. No significant correlation was found between EPDS and MAAS-intensity, ZSDS and MAAS-intensity/global, and POMS-D and MAAS-intensity [45]. Another study found that higher depression scores were associated with lower MAAS-quality and MAAS-global scores, but not MFAS-total or MAAS-intensity [80]. Goecke et al. [84] found a negative correlation between EPDS and the quality but not the intensity of MFA in the third trimester and at three weeks postpartum, in addition to global MFA at three weeks postpartum. Seimyr et al. [98] did not find a correlation between depression and MFAS-global, but found that higher depression was associated with two subscales of the MFAS–higher IV (experience of fetal movement) and lower V (positive experiences of pregnancy). Similarly, Barone et al. [82] found that women who scored higher on the fantasy and sensitivity subscale of the PAI reported higher depression, however total MFA was not associated with depression. These results highlight the multifaceted nature of MFA as a construct, and the limitations of employing a variety of screening tools across studies. This raises the question of whether MFA should be continued to be measured as a global construct, or as a set of factors.

#### Depression and postpartum bonding

In four out of five studies that investigated depression and early postpartum bonding (defined in this review as up to 12 weeks after childbirth), higher depression was associated with lower bonding after childbirth [27, 53, 95, 96]. No significant finding was reported in the remaining study [80]. This suggests a continued effect of low mood on a mother’s ability to bond with and interact with her baby, even after the antenatal period.

#### Anxiety and MFA

Ten studies examined anxiety in relation to MFA. Five of these studies used the MAAS and found that higher anxiety was associated with lower MAAS-quality [45, 86, 92, 97, 99]. No correlation was found between anxiety and MAAS-intensity in four of those studies [45, 86, 97, 99]. This suggests that anxiety may have an effect on the closeness rather than the strength of the MFA. Two of these studies found no correlation between MAAS-global and anxiety [45, 86], one reported a positive correlation [92] and the remaining two studies did not report on MAAS-global scores [97, 99]. Figueiredo and Costa [27] found that poorer MFA predicted higher postpartum anxiety but not antenatal anxiety. Rubertsson et al. [46](p156) found that higher anxiety was associated with higher ‘anticipation’ (“dreams, fantasies and future plans for the baby”) but not ‘interaction’ (“mother’s feelings for her baby and sharing her experience with others”) or ‘differentiation’ (“knowledge about the baby’s personality and attributes”) on the PAI-R. No association was found between MFA and pregnancy-related anxiety [89] or anxiety disorders [93] or anxiety when using the MFAS as a measure of MFA [81].

#### Anxiety and postpartum bonding

Two studies investigated anxiety and mother-infant bonding in the early postpartum period. One study, which included a maternal age range from 16 to 40 years, found that anxiety was associated with poorer bonding, characterized by stronger negative emotions towards and lower emotional involvement with the baby [27]. Similarly, higher state and trait anxiety was correlated with lower postpartum bonding [81].

#### Stress and MFA

Three studies investigated stress in relation to MFA. Higher pregnancy-specific stress was correlated with stronger MFA suggesting that a reallocation of resources towards the baby and the maternal role may be associated with greater sensitivity towards the baby’s needs and a richer bonding experience [83, 88]. In contrast, a negative association was observed between life stress and MFA suggesting that external stressors and negative life events may take away resources from the mother that may have been devoted to the development of MFA [88]. A positive correlation was observed between the quality and intensity of MFA in women with a ‘positive appraisal’ coping style [99]. Similarly, higher MFA was associated with lower use of emotion-focused coping and a willingness to seek support when required, however this pattern was only observed in the first trimester [94].

#### Interpersonal relationships and MFA

Six out of seven studies investigating MFA and social support found that higher MFA was associated with greater social support [45, 53, 73, 83, 87, 97]. In contrast to Schmidt et al. [97], who found a positive correlation between the quality of the MFA and social support, White et al. [99] found no significant correlation. Social support was not found to be correlated with the intensity of the MFA [97, 99].

In all six studies investigating partner support and MFA, a good intimate partner relationship was associated with stronger MFA [45, 46, 81, 82, 90, 92]. MFA was greater in women with higher perceived couple adjustment [81, 82, 92], higher emotional and instrumental spousal support [90], and lower control, domination and criticism within the intimate partner relationship [45]. Higher partner support was associated with greater endorsed feelings towards the baby and sharing of pregnancy experiences with others, but neither of the two other PAI-R subscales or PAI-R global score [46].

These findings are consistent with previous research suggesting that social support can act as a protective factor when individuals are faced with stressful and challenging situations [100]. In the transition to motherhood, interpersonal and partner support may allow women to share the rewarding experiences of pregnancy with another person [73], facilitate planning and imagination of the child’s future [82] and allow for better adjustment to motherhood [90].

#### Interpersonal relationships and postpartum bonding

One study examined the relationship between social support and postpartum bonding. In this study, positive associations between social support and MFA observed during pregnancy continued into the postpartum period in relation to bonding [53].

#### Other domains of mental health/wellbeing and MFA

Two studies examined the impact of wellbeing on MFA. Higher psychological wellbeing ratings were associated with higher global and quality MFA scores on the MAAS [84]. Greater wellbeing was correlated with higher MFA (on the MFAS) and lower distress in the first trimester, but not the remaining trimesters [94]. Neither body dissatisfaction [85] nor disordered eating [90] were found to be correlated with MFA.

#### Other domains of mental health/wellbeing and postpartum bonding

No studies included in this review investigated stress, wellbeing, body dissatisfaction or disordered eating in relation to early postpartum bonding.

#### Patterns across the antenatal and postnatal periods

The longitudinal studies included in this review were examined for patterns of continuity across the antenatal and postnatal periods. Despite their differences regarding the theoretical processes involved in MFA and postpartum mother-infant bonding, three studies highlighted the relationship between the constructs across the perinatal period. Doster et al. [81] found that higher MFA was positively correlated with postpartum bonding. Figueiredo and Costa [27] found that lower MFA predicted poorer postpartum bonding at three months. Similarly, Rubertsson and colleagues [46] found that women who reported fewer positive feelings about birth and the early postpartum period during their pregnancy also reported lower MFA. Other studies found that women’s mental health and wellbeing during pregnancy had an influence on their functioning postpartum. For example, one study found that women with fewer supportive people during pregnancy showed higher depression and lower bonding postpartum [53]. Another study showed that MFA predicted mood not only in late pregnancy but also at five days postpartum [95]. Women who reported continuous depressive symptoms during pregnancy and up to one month postpartum showed sustained bonding difficulties with their babies throughout pregnancy and the early postpartum period [96].

### Additional findings

#### Prevalence rates

A number of studies reported on the percentage of women who scored above the cut-off for elevated depression and anxiety. Prevalence rates of depression were reported in 11 studies, and ranged from 9–59% (M = 27.29, SD = 19.32) [27, 45, 73, 80, 82, 86, 87, 91, 94, 96, 98]. Prevalence rates of anxiety were reported in three studies, and ranged from 25–36% (M = 31.40, SD = 4.67) [27, 86, 94]. Given the disparity in assessment measures used and varying ways of reporting on MFA, the prevalence of good versus poor MFA was unable to be calculated.

#### Demographic variables and MFA

An examination of the role of demographic variables in relation to MFA also produced mixed findings (results presented in *Table 5*). Seven of the 25 included studies did not address the role of any demographic variables. Education and primiparous/multiparous status were the most frequently examined variables. Out of 15 studies investigating the role of maternal age, seven found that older mothers reported lower MFA while the remaining six studies found no significant relationship. Six out of 10 studies examining gestational age found that women further along in their pregnancies reported stronger MFA. The remaining four studies found no significant relationship. Socioeconomic status was evaluated in relation to MFA in six studies, none of which reported a significant effect. Two out of seven studies assessing the role of women’s relationship status found that women who were married or in a de facto relationship reported higher MFA. Higher maternal education was associated with lower MFA in three out of 11 studies, with the remaining eight studies reporting no significant effect of education. Two out of three studies found a positive relationship between employment status and MFA. Three studies found that women with planned pregnancies reported stronger MFA, while an additional two studies found no significant effect. Five out of 11 studies found that primiparous women reported higher MFA scores than multiparous women. Overall, findings about the interaction between demographic factors and MFA were variable and under-reported, highlighting the need for further research in this area.

**Table 5:** *MFA* and demographic variables.

Article	Maternal age	Gestational age	SES	Relationship status	Education	Employment	Planned pregnancy	Primiparous	Other
1	n/a	n/a	No	No	n/a	n/a	n/a	n/a	
2	No	Yes (+)	n/a	No	No	No	n/a	No	
3	n/a	n/a	n/a	n/a	n/a	n/a	n/a	n/a	
4	No	n/a	No	n/a	n/a	n/a	Yes (+)	No	Number of children (-)
	No	n/a	No	n/a	No	n/a	n/a	No	
5	n/a	n/a	n/a	n/a	n/a	n/a	n/a	n/a	
6	n/a	n/a	n/a	n/a	Yes (-)	n/a	n/a	n/a	
7	No	Yes (+)	n/a	No	No	n/a	n/a	Yes (+)	
8	Yes (-)	No	n/a	n/a	No	n/a	No	n/a	
9	n/a	n/a	n/a	n/a	No	Yes (+)*	n/a	n/a	*Strongest relationship with stay at home caregiver, followed by full-time work, then part-time work
10	No	No	No	n/a	No	n/a	No	Yes (+)	Attendance at prenatal classes (+)
11	n/a	n/a	n/a	n/a	n/a	n/a	n/a	n/a	
12	No	Yes (+)	No	n/a	No	n/a	n/a	n/a	
13	n/a	n/a	n/a	n/a	n/a	n/a	n/a	n/a	
14	Yes (-)	Yes (+)	No	Yes (+)	Yes (-)	n/a	n/a	No	
	No	Yes (+)*	n/a	Yes (+)**	No	n/a	Yes (+)	Yes (+)***	*MAAS-total and MAAS-intensity **MAAS-total and MAAS-quality ***MAAS-intensity Period after fetal movement detected (+)
15	Yes (-)	No	n/a	n/a	n/a	n/a	n/a	No	
16	n/s	n/a	n/a	n/a	n/a	n/a	n/a	n/a	
17	n/a	n/a	n/a	n/a	n/a	n/a	n/a	n/a	
18	n/a	n/a	n/a	n/a	n/a	n/a	n/a	n/a	
19	n/a	n/a	n/a	n/a	n/a	n/a	n/a	n/a	
20	Yes (-)*	n/a	n/a	n/a	Yes (-)*	n/a	n/a	Yes (+)*	*PAI-Anticipation and PAI-Interaction
21	Yes (?)	Yes (+)	n/a	n/a	n/a	n/a	n/a	n/a	
22	Yes (-)*	n/a	n/a	No	No	Yes (+)**	n/a	Yes (+)	*MFAS-IV **MFAS-III and IV
23	No	No	n/a	No	No	n/a	Yes (+) *	No	*MAAS-Intensity

#### Demographic and mental health variables

Over half of the included studies (n = 14) did not examine the potential role of demographic factors in relation to domains of mental health or wellbeing (results presented in *Table 6*). Multiparous status and having a higher number of children were associated with higher depression in three studies. All four of the studies examining depression and gestational age reported no significant findings. Given the low number of studies investigating domains of mental health other than depression (i.e. anxiety, stress, body dissatisfaction, couple adjustment) and the even fewer studies that examined these domains in relation to demographic factors, no trends could be identified.

**Table 6:** Mental health constructs and demographic variables.

Article	Variable	Maternal age	Gestational age	SES	Relationship status	Education	Employment	Planned pregnancy	Primiparous	Other
1	Depression, social support	n/a	n/a	n/a	n/a	n/a	n/a	n/a	n/a	
2	Depression	No	No	n/a	n/a	n/a	n/a	n/a	n/a	
	Couple adjustment	Yes (-)	No	n/a	n/a	n/a	n/a	n/a	n/a	
3	Pregnancy stress	No	No	No	No	No	No	No	Yes (+)	
4	Depression	n/a	n/a	n/a	n/a	n/a	n/a	n/a	n/a	
	Depression, anxiety, partner relationship	n/a	n/a	n/a	n/a	n/a	n/a	n/a	n/a	
5	Depression, anxiety	n/a	n/a	n/a	n/a	n/a	n/a	n/a	n/a	
6	Depression	n/a	n/a	n/a	n/a	Yes (-)	n/a	n/a	n/a	History of miscarriage (+)
7	Depression	n/a	No	n/a	n/a	n/a	n/a	n/a	n/a	
8	Depression	No	No	n/a	n/a	n/a	n/a	n/a	n/a	
9	Depression	n/a	n/a	n/a	n/a	n/a	n/a	n/a	n/a	
10	Stress	No	No	No	n/a	No	n/a	No	No	
11	Depression	n/a	n/a	n/a	n/a	n/a	n/a	n/a	n/a	
12	Anxiety, social support	n/a	n/a	n/a	n/a	n/a	n/a	n/a	n/a	
13	Body dissatisfaction	No	n/a	n/a	n/a	Yes (+)	No	n/a	n/a	
14	Depression	Yes (-)	No	Yes (-)	Yes (+)	Yes (-)	n/a	n/a	Yes (+)	High-risk pregnancy (+), ethnicity (+)
	Depression, anxiety, partner relationship	n/a	n/a	n/a	n/a	n/a	n/a	n/a	n/a	
15	Depression	No	n/a	No	Yes (-)	n/a	n/a	n/a	No	Number of children (+), pregnancy complications (+)
16	Depression, anxiety	n/a	n/a	n/a	n/a	n/a	n/a	n/a	n/a	
17	Depression, social support	n/a	n/a	n/a	n/a	n/a	n/a	n/a	n/a	
18	Depression	n/a	n/a	n/a	n/a	n/a	n/a	n/a	n/a	
19	Depression	n/a	n/a	No	n/a	No	n/a	n/a	n/a	
20	Depression, anxiety	n/a	n/a	n/a	n/a	n/a	n/a	n/a	n/a	
21	Depression, depressive rumination, anxiety	n/a	n/a	n/a	n/a	n/a	n/a	n/a	n/a	
22	Depression	n/a	n/a	n/a	n/a	n/a	n/a	n/a	n/a	
23	Depression, anxiety, stress	n/a	n/a	n/a	n/a	n/a	n/a	n/a	n/a	

When considering distress as a general construct, two studies found an effect of age, such that older women reported higher distress. However, the remaining six studies examining the role of age found no significant relationship. None of the studies examining gestational age (n = 6), employment (n = 2) or planned/unplanned pregnancy status (n = 2) found a significant effect for distress. Four out of five studies found no relationship between SES and distress. Conflicting findings were observed for relationship status (n = 3), education (n = 5) and primiparous or multiparous status (n = 4). Further research is required to facilitate increased understanding of the role of personal and contextual variables in relation to maternal mental health.

## Discussion

This review sought to systematically analyze the literature surrounding MFA, early postpartum bonding and maternal mental health in the antenatal and early postnatal periods, in order to clarify whether a relationship exists between variables. Our review found mixed results as to the association between MFA/postpartum bonding and various domains of mental health. The review identified a number of gaps within the current literature pertaining to the measures employed within studies for antenatal populations, theoretical understanding of MFA, and data collection points during the antenatal and postnatal periods.

### Is there a relationship between mental health, MFA and postpartum bonding?

This review aimed to determine whether relationships existed between a number of mental health domains and both MFA and early postpartum bonding. Consistent findings were observed for depression and interpersonal relationships in the antenatal and postnatal periods. However, due to discrepancies in study findings and a small number of studies examining particular variables, no patterns could be identified for anxiety, stress, body dissatisfaction, disordered eating, depressive rumination or subjective wellbeing. Further research is required in these areas.

Depression was the most studied mental health variable within the included studies. Depression was associated with lower MFA and postpartum bonding in the majority of publications. These findings are supportive of the claim that maternal mood negatively impacts on a mother’s ability to bond with her baby both during pregnancy and in the early postpartum period [45, 86]. Despite these findings, some discrepancies were noted including four studies with non-significant results. All four of these studies included only one time point in pregnancy or employed cross-sectional designs spanning across trimesters of pregnancy. One explanation for the non-significant results may be the variation in gestational age [85] and the assessment of MFA early in pregnancy before fetal movement could be detected [87]. This supports the idea that the nature of a mother’s attachment towards her baby may change as she moves throughout her pregnancy and highlights the need to avoid generalizing results from one trimester to another [85, 87]. Additional explanations for this include differences in participant samples, discrepancies in data collection points and variations in screening tools used to assess depression and MFA.

Although less studied than depression, positive interpersonal relationships were associated with better MFA and postpartum bonding outcomes. Six out of seven studies examining social support and all six studies investigating intimate partner relationships found associations with higher MFA. Similarly, one study reported a positive correlation between social support and postpartum bonding. These findings are consistent with previous research citing interpersonal support as a potential buffer for stress, isolation and maladaptive adjustment to motherhood [73, 90, 100].

### Study design and methodology

A strength of most studies in the review was the employment of diverse samples. Participants were aged 16–45 years, from 13 countries, with an average sample size of 239. Participants included women from lower socioeconomic backgrounds, community and hospital samples, of primiparous and multiparous status, with diagnosed mental illnesses, and women who had conceived with and without assisted reproductive technology. Future studies should continue to utilize diverse groups to maximize the generalizability of results and yield clinical insights into difficulties faced by higher risk groups. Specifically, research into younger and older mothers would highlight specific developmental and parenting challenges that may impact the mother-infant relationship during and after pregnancy.

A weakness in the included studies was the way in which data was collected. Less than half of the studies employed longitudinal designs. Although cross-sectional data has many benefits including low-cost, efficiency of data collection and low participant burden [101], reliance on cross-sectional data impeded analysis of the changing mother-baby relationship over time. Further, there was wide variation in the time points (e.g., 9 weeks, 24–28 weeks) and time brackets (e.g., 2–40 weeks gestation, first half of pregnancy) used in data collection. Future research efforts should focus on identifying appropriate standardized points of data collection so that researchers are able to synthesize findings across studies to identify patterns and trends.

### Is there a relationship between mental health and MFA?

Consistent with previous reviews in the field of antenatal mental health [102], the primary mental health focus of the included articles was depression. There was evidence that lower depression scores were associated with higher MFA, a finding supported in 15 out of 19 studies. The findings surrounding anxiety and stress were mixed, and there was insufficient research on rumination, disordered eating or body dissatisfaction to identify broader trends. Good partner and social relationships were consistently related to better MFA. Although less attention has been paid to positive affect within the literature [102], the studies which focussed on positive affect (e.g. wellbeing, social support, partner relationship) produced more consistent findings. Possible explanations for these findings are considered in more detail below.

### Use of diverse generic domain mental health measures

The included studies employed 34 different instruments to assess 11 domains of mental health. Twenty-eight of the 34 screening tools used were domain-generic (i.e. not pregnancy-specific). Use of general measures for a specialized population reduces reliability and validity [103], and may result insufficient attention being paid to the unique features of maternal populations [104, 105]. This problem could be addressed by either validating existing generic domain measures for use in pregnancy and the postpartum period, and recognising their limitations when interpreting results, or developing pregnancy and postpartum-specific measures to ensure greater sensitivity to the unique experiences of pregnancy [104, 106]. Many of the instruments used have not been validated for use in pregnancy and in the postpartum period–a factor that may explain some of the variability in observed results. An exception to this was the use of the EPDS in 11 of the studies. The EPDS is cited as the most widely used screening tool for antenatal depression [107] and has been validated for maternal populations [108]. These two patterns support Mogos et al.’s [104] ^(p219)^ assertion that there is a lack of “valid, reliable and responsive” instruments developed for use in maternal populations.

### The construct of MFA and how it is being measured

The current review exemplified the existing tensions within the literature regarding the lack of consensus surrounding the definition and theoretical underpinnings of the MFA construct [82, 87, 109, 110] and the way it should be measured [44, 111]. This was reflected through the different ways in which the emerging relationship between mother and baby were described (i.e. MFA, prenatal attachment, antenatal attachment, maternal attachment, perinatal bonding and emotional involvement), and the different screening tools used to measure MFA (n = 12). In addition to impeding a comparative analysis of study findings, inconsistent terminology and screening instruments may contribute to a further divide between theoretical schools of attachment/bonding and hinder attempts to consolidate a strong theoretical foundation.

There was a consensus within the included articles and the wider literature about the uniqueness of the mother-fetal relationship [84, 86, 111], as distinct from postpartum mother-infant bonding [112]. The findings of our review support Barone et al.’s [82] suggestion that researchers need to cease interpreting global MFA scores in isolation, and investigate the individual subscale scores. This idea is exemplified in Condon and Corkindale’s [45] suggestion of distinguishing the quality (the closeness of the relationship) and intensity (the strength of the preoccupation with the baby) of MFA. Our systematic review found that when studies employed Condon’s MAAS, the quality of the MFA was consistently related to maternal mental health, whereas the intensity was not. Previous research has identified a possible reason for this finding as the role of external factors (e.g. life events, stressors, family situation, work commitments), as opposed to internal factors (e.g. mental health) as influencing MFA intensity [45, 82]. From a theoretical standpoint, these findings support the notion of MFA as a multidimensional construct [112]. From a research perspective, these findings support the use of both subscale and global scale scores [82, 113].

### Capturing the whole picture (a holistic approach)

A final trend that emerged within the current review was a failure to conceptualize studies that followed women throughout the entirety of the pregnancy and postpartum period. Cross-sectional designs accounted for more than half of the studies within the review despite the strong empirical evidence for the changing course of maternal mental health [74] and attachment [42, 114] across the maternity continuum. The majority of articles considered for review had a solely antenatal *or* postnatal focus. As previously recognized [74, 107], there continued to be a focus on postpartum outcomes, and a neglect of antenatal processes. This was reflected in studies investigating the relationship between antenatal wellbeing/mental health and postpartum bonding, but not MFA (despite following women throughout their pregnancies)–a pattern that resulted in five studies being excluded at the full-text review [115–118].

A richer and more holistic account of the changing wellbeing trajectory requires longitudinal studies that span across pregnancy and the early postpartum period. Such studies are not only desirable, but feasible because of pregnant women’s altruistic attitudes about participating in research [119], low attrition rates [27, 46] and intensive contact with medical professionals [102]–three factors which make pregnant women ideal candidates for longitudinal research [120].

A further limitation of the current literature was the failure to control for the potential effect of demographic variables on the relationship between wellbeing and MFA. Eighteen out of the included 25 studies considered demographic factors in relation to MFA, while only 11 considered demographics in relation to wellbeing or mental health. As a result, we were unable to derive patterns from the data as to the effect of factors such as maternal age, socioeconomic status, level of education and pregnancy history. Failure to consider these contextual variables within individual study analyses may contribute to the conflicting findings identified here, which will in turn continue to limit our understanding of the relationship between MFA and maternal wellbeing.

### Limitations

A weakness in any systematic review is that the interpretation of the findings is dependent on the quality and scope of the included studies. A specific limitation for this review is the lack of screening tools validated for use in antenatal populations, which makes it difficult to draw conclusions about best practice in the selection of measures. Secondly, there was an overwhelming reliance on self-report questionnaire data (as opposed to clinical, diagnostic assessment), which may have produced underreporting or overestimation of symptomatology, and associated bias. However, this approach remains valuable given the sensitive nature of information asked, low participant burden, and practicality of data collection. Thirdly, given that pregnancy is a fluid and changing time, the absence of standard data collection points may mean that results are overgeneralized. We acknowledge that only two studies examined MFA in relation to young mothers (those under 18 years), and that consequently our findings cannot be generalized to this group. Finally, although we completed a systematic search of the relevant literature, it is possible that we screened out or failed to include potentially relevant publications.

### Implications for future research

The findings of this review support four important considerations for future research. First, there is a need to validate mental health measures for use in antenatal populations, or alternatively develop new measures specifically for pregnant women. Second, continued efforts must be made to standardize data collection points during pregnancy and postpartum with culturally, socioeconomically, and geographically diverse samples where possible, to maximize the generalizability of findings. Third, a consensus must be made in relation to the terminology used to describe MFA, and a renewed commitment to theorizing the construct. Finally, we need to recognize the limitations of focusing exclusively on the postpartum period, and the value of longitudinal studies based on a more holistic conception of the total pregnancy and postpartum period.

### Conclusion

This systematic review highlights a number of gaps within the current literature that need to be addressed before the relationship between maternal mental health and MFA can be better understood. Methodologically rigorous longitudinal studies that span the full pregnancy and postpartum period with diverse participant samples will enable researchers to more clearly understand the role that maternal wellbeing and mental health play in the development of MFA and the bonding relationship between mother and baby. Given that only a minority of women with mental health difficulties receive treatment [121], and the strong empirical support for the negative effects of poor maternal mental health for both mother and infant [122], further research in this area is critical. Improved understanding of this relationship will support more accurate identification of at-risk mothers and the development and implementation of appropriate interventions.

## Supporting information

S1 Table PRISMA checklist. (DOCX)
